# Supplementary material for: Qualitative Study to Explore the Occupational and Reproductive Health Challenges among Women Tobacco Farm Laborers in Mysore District, India
Source: Int J Environ Res Public Health. 2024 May 9;21(5):606. doi: 10.3390/ijerph21050606 (PMC11121108; doi:10.3390/ijerph21050606)
Supplement: Supplementary file 1 [file ijerph-21-00606-s001.zip › ijerph-2971653-supplementary.pdf]

Supplemental Material 1 - **Interview questions**

A. Demographics

	Question	Response	
1.	Age (in years)		
2.	Date of Birth		
3.	Place of Birth, district and State		
4.	What is your marital status?	Single	1
		Married	2
		Widow	3
		Separated/divorced	4
		Others, specify	5
		Refused	99
5.	What religion do you follow?	Hindu	1
		Muslim	2
		Sikh	3
		Christian	4
		Jain	5
		Buddhism	6
		No religion	7
		Others (specify)	8
		No response	9
		Refused	99
6.	Do you belong to particular caste?	Scheduled Caste	1
		Scheduled Tribes	2
		Other Backward Caste	3
		General Caste	4
		Refused	99
7.	Years of Education		
8.	Level of education	Professional degree/Post graduate	1
		Graduate degree	2
		Secondary school /Intermediary	3
		High school (class VI to X)	4
		Primary School (upto Class V)	5
		*Literate, no formal education	6
		**Illiterate	7
		Refused	99
9.	What is your total annual Household income?	Rs.	

B. History of tobacco farming:

	Activity	Yes	No	How many hours in a day do you work?	How many years do you do this work?
1.	Cultivation				
2.	Harvesting				
3.	Curing/ drying				
4.	Sorting				
5.	Stacking				
6.	Packing				

C. History of tobacco use:

1.	Have you ever used tobacco in any form (Smoking, chewing, snuff, etc)?	Yes 1<br>No 2		If no go to Q.9
2.	In what forms have you consumed tobacco?	Smoking 1<br>Smokeless 2		
3.	At what age did you first started smoking regularly?	…………… in years		
4.	At what age did you first started using smokeless tobacco regularly?	…………… in years		
5.	Do you currently consume tobacco?	Yes 1<br>No 2		
6.	Have you consumed tobacco in the last 12 months? (past user)	Yes 1<br>No 2		
7.	Quantity and duration of use (both current and past users)			

Type of tobacco used		Frequency per day	Duration of use	If Quit, since	
				Years	Months
Smoking Tobacco	Bidi				
	Cigarette				
	Cigar/Hukka/Chillum/Pipe				
Smokeless Tobacco	Khaini				
	Gutka				
	Chewing tobacco				
	Pan with Zarda				
	Snuff				
	Oral tobacco – Gul, Kiwam				
Arecanut without tobacco	Pan without zarda				
	Supari				

8.	Are you exposed to tobacco smoke from others?<br>(e.g. at home, at workplace, while travelling, any other place)	Yes 1<br>No 2
9.	Did you consume tobacco when you were pregnant?	Yes 1<br>No 2<br>Occasionally 3

D. Occupational and reproductive health:

Total number of pregnancies: …………

History of abortion / miscarriage / death of child:

a) Atleast one abortion: Yes / No / Don't know / Refuse
b) Miscarriage: Yes / No / Don't know / Refuse
c) Death of child: Yes / No / Don't know / Refuse

	Questions	Yes	No	Don't know	Refuse to answer
1.	Have you ever been involved in tobacco farming when you were pregnant?				
2.	Have you ever been exposed to tobacco dust when you were pregnant?				
3.	Do you use protective clothing when handling pesticides during pregnancy?				
4.	Did you develop any allergies during your pregnancy?				
5.	Did you develop any allergies after child birth?				
6.	Did your child develop any allergies or respiratory problems within 6 months of birth?				
7.	Did your child develop any allergies or respiratory problems within 1 year of birth?				

Supplemental Material 2

Focus group discussion question guide based on social ecological model

**Socioecological model**	**Question**	**Prompts**
Individual	How is your health? Do you think you are healthy?	How is your reproductive health? (Periods, pregnancy or menopause)
	How was your pregnancy and child birth?	What type of farming work did you do during pregnancy?
	Can you talk about the personal protection like gloves, boots, mask use while handling tobacco? When you use these?	How often do you to get access to these?
	What do you know about the effects of tobacco use during pregnancy and lactation?	What was the source of these information?
	What were the challenges you faced during your periods by working in tobacco farms?	
	What were the challenges you faced during your pregnancy by working in tobacco farms?	
	What type of problems do you think could occur in pregnancy due tobacco use?	
Interpersonal	What are your thoughts about tobacco dust and/or smoke around you during pregnancy?	
	What type health problems are there in your family?	How often do you go to doctor for this problem?
	What type of support did you receive from you family and friends during pregnancy?	What type of support you wish you had?
Organisational	What type of support or services did you receive from your farming organisation during your pregnancy and child birth?	What type of resources you wish you had?
	What type of tobacco cessation programs or recourses are available to you?	What type of resources you think is missing?
Community	Can you elaborate on the maternity benefits you received from your community?	What were the challenges in receiving the benefits?
Policy	To your knowledge, what type of policies or programs are there for women tobacco farmers?	What type of policies do you think is required?
Closing question	Is there anything else you would like to share with me?	About your health?<br>About your family and community?
